# Sedentary songbirds maintain higher prevalence of haemosporidian parasite infections than migratory conspecifics during seasonal sympatry

**DOI:** 10.1371/journal.pone.0201563

**Published:** 2018-08-22

**Authors:** Samuel P. Slowinski, Adam M. Fudickar, Alex M. Hughes, Raeann D. Mettler, Oxana V. Gorbatenko, Garth M. Spellman, Ellen D. Ketterson, Jonathan W. Atwell

**Affiliations:** 1 Department of Biology, Indiana University, Bloomington, IN, United States of America; 2 Environmental Resilience Institute, Indiana University, Bloomington, IN, United States of America; 3 School of Natural Sciences, Black Hills State University, Spearfish, SD, United States of America; 4 Zoology Department, Denver Museum of Nature and Science, Denver, CO, United States of America; Universidade Federal de Minas Gerais, BRAZIL

## Abstract

Long-distance migrations influence the physiology, behavior, and fitness of migratory animals throughout their annual cycles, and fundamentally alter their interactions with parasites. Several hypotheses relating migratory behavior to the likelihood of parasitism have entered the literature, making conflicting, testable predictions. To assess how migratory behavior of hosts is associated with parasitism, we compared haemosporidian parasite infections between two closely related populations of a common North American sparrow, the dark-eyed junco, that co-occur in shared habitats during the non-breeding season. One population is sedentary and winters and breeds in the Appalachian Mountains. The other population is migratory and is found in seasonal sympatry with the sedentary population from October through April, but then flies (≥ 900 km) northwards to breed. The populations were sampled in the wild on the shared montane habitat at the beginning of winter and again after confining them in a captive common environment until the spring. We found significantly higher prevalence of haemosporidian parasite infections in the sedentary population. Among infected juncos, we found no difference in parasite densities (parasitemias) between the sedentary and migrant populations and no evidence for winter dormancy of the parasites. Our results suggest that long-distance migration may reduce the prevalence of parasite infections at the population level. Our results are inconsistent with the migratory exposure hypothesis, which posits that long-distance migration increases exposure of hosts to diverse parasites, and with the migratory susceptibility hypothesis, which posits that trade-offs between immune function and migration increase host susceptibility to parasites. However, our results are consistent with the migratory culling hypothesis, which posits that heavily infected animals are less likely to survive long-distance migration, and with the migratory escape hypothesis, which posits that long-distance migration allows host populations to seasonally escape areas of high infection risk.

## Introduction

Long-distance migration is found in all major animal groups [1]. Animals likely evolved long-distance migration to track seasonal resources (reviewed in [2]), avoid harsh environmental conditions, and/or reduce predation risk [3]. Regardless of their evolutionary origins, long-distance migrations influence the physiology, behavior, and fitness of migratory animals throughout their annual cycles, and fundamentally alter their interactions with pathogens and parasites. As migratory animals move seasonally across the landscape, entirely new spatial and temporal dynamics emerge with respect to host-parasite interactions. This adds complexity to our theoretical frameworks and empirical characterizations of critically important topics in both disease ecology and host-parasite coevolution. In the present study, we leveraged intra-specific variation in migratory strategies to examine how the migratory behavior of animal hosts relates to the prevalence, intensity, and diversity of their parasites, and to the timing of parasite life cycles.

Several non-mutually exclusive hypotheses have been put forth to predict the association between long-distance host migratory behavior and the dynamics of parasite infections, including their prevalence, diversity, and virulence (reviewed in [4]). According to the migratory culling hypothesis, heavily infected animals are less likely to survive the physiological stresses of long-distance migration [5]. Consequently, infected animals are predicted to be culled from host populations via natural selection. The migratory escape hypothesis posits that long-distance migration allows host populations to seasonally escape areas that have become heavily infested with parasites [6]. Hence the migratory culling hypothesis and the migratory escape hypothesis both predict that parasite prevalence should be higher in sedentary host populations than in closely related migratory host populations. This prediction has received some empirical support from studies on both migratory vertebrates and invertebrates [7–9].

On the other hand, the migratory exposure hypothesis postulates that migrating through multiple different habitats increases parasite exposure in migratory animals. Migratory animals may be exposed to different parasites at their breeding grounds, stopover sites, and wintering grounds, and consequently accumulate a higher prevalence and diversity of parasites than sedentary animals [10]. Furthermore, the migratory susceptibility hypothesis suggests that trade-offs between migration and immunity could increase the susceptibility of migrants to acquiring new infections during the course of migration, leading to an even higher prevalence of parasites in migrants relative to sedentary animals (reviewed in [11]). See Table 1 for a summary of hypotheses and predictions about how long-distance host migration should be associated with parasite prevalence.

**Table 1:** A summary of the hypotheses and predictions discussed in the present study.

Hypothesis	Predicted parasite prevalence and diversity
**Migratory culling**	**Sedentary prevalence > Migrant prevalence**
**Migratory escape**	**Sedentary prevalence > Migrant prevalence**
**Migratory exposure/Migratory susceptibility**	**Migrant prevalence > Sedentary prevalence**
**Migratory exposure/Migratory susceptibility**	**Migrant parasite diversity > Sedentary parasite diversity**

In support of the migratory exposure hypothesis, several studies have found that migratory species of birds harbor a higher diversity of parasites relative to non-migratory species [12–15]. While such among-species comparisons suggest that host migration may increase exposure to parasites and infection risk, fewer studies have made comparisons within vertebrate species, where the interpretive challenges of phylogenetic history and host-specificity should be minimized. In the only published study that we are aware of comparing parasite prevalence with migratory propensity *within* a vertebrate host species, Kelly, MacGillivray [16] found a higher prevalence of haematozoan parasite infections in adult long-distance migrant song sparrows *Melospiza melodia* relative to conspecifics that migrated shorter distances. More research is needed to assess how migratory behavior is associated with parasitism among closely related populations *within* vertebrate species. In the present study, we compared haemosporidian parasite infections in a sedentary songbird population versus a closely related conspecific migrant population. Haemosporidians are protozoan parasites that infect vertebrate red blood cells and are transmitted by dipteran insect vectors. Haemosporidians include the malaria-causing genus *Plasmodium*. Avian haemosporidian parasites can be pathogenic in wild populations (e.g. [17–19]). Haemosporidian parasites have been studied extensively in avian populations, and serve as a model for the study of ecological and evolutionary dynamics of host-parasite interactions (reviewed in [17]). We assess how host migratory strategies are associated with the prevalence and intensity (objective 1), and the timing of seasonal dormancy (objective 2) of haemosporidian parasites.

### Objective 1: Association between host migration and infection prevalence and parasitemia

Our first objective was to explore how variation in host migratory behavior is associated with the prevalence and parasitemia of a widespread and diverse class of parasites, the haemosporidia, that infect a common songbird host, the dark-eyed junco (*Junco hyemalis*). Specifically, we compared haemosporidian parasite infections in the bloodstream, during seasonal sympatry, between a sedentary junco population and a closely related migrant junco population that share wintering grounds in Virginia, USA. Based on the migratory culling hypothesis and the migratory escape hypothesis, we predicted that the prevalence and/or parasitemia of haemosporidian parasite infections would be higher in the sedentary junco population than in the migrant population (Table 1). Alternatively, based on the migratory exposure hypothesis and the migratory susceptibility hypothesis, we predicted that the prevalence and/or parasitemia of haemosporidian parasites would be higher in the migrant population than the sedentary population (Table 1).

### Objective 2: Measuring seasonality in the parasite lifecycle

In temperate climates, some avian haemosporidian parasites alternate between an active stage in the spring and summer, during which the parasites grow and replicate and produce transmissible life stages in the bloodstream of their host, and a dormant stage in the winter during which the parasites sequester in the host organs (reviewed in [17]). Thus, our second objective was to examine whether haemosporidian parasites in our study system exhibit this seasonal pattern. We predicted that prevalence and parasitemia of parasites detectable in the host blood stream would be lower during the winter (when parasites were dormant in the host organs) and would increase in the spring (when parasites re-emerged and started replicating in the host blood stream). We tested these predictions by measuring haemosporidian prevalence and parasitemia in blood samples collected from juncos from both populations during the winter (December) and spring (early March and late March).

## Materials and methods

### Study system

We compared haemosporidian parasite infections between two populations of dark-eyed junco hosts that share wintering habitat at the Mountain Lake Biological Station in Pembroke, VA (37.37°N, 80.52°W). Juncos in one of the populations (the sedentary population, *Junco hyemalis carolinensis*) are generally year-round residents, breeding in Virginia during the spring and summer, with most individuals remaining near their territories during the non-breeding season [20]. In contrast, the other population (the migrant population, *Junco hyemalis hyemalis*) co-occurs with *J*. *h*. *carolinensis* in Virginia during the non-breeding season, but migrates northwards (≥ 900 km to Canada, Northern New England, and/or Alaska) to breed [20, 21]. The sedentary and migrant junco subspecies are thought to have diverged within the last 15,000 years, since the last major glaciation [22]. Our field site in Virginia lies far South of the breeding range of the migratory subspecies [20], and the sedentary and migrant junco subspecies have never been observed to hybridize at our field site, despite extensive, long-term monitoring efforts.

### Bird capture, and sampling in the field

The details of the capture and housing methods used in this study have been described previously [23]. Briefly, sedentary (*Junco hyemalis carolinensis*, *n = 19*) and migrant (*Junco hyemalis hyemalis*, *n = 18*) male juncos were captured from 4–12 December 2013, at the Mountain Lake Biological Station in Giles County, Virginia (37.37°N, 80.52°W). Juncos were captured in mist-nets and potter traps baited with cracked corn and millet. Population status (sedentary or migrant) was determined based on bill coloration, plumage, and wing chord [20]. Age class was determined for each bird based on wing plumage color [20], and the distribution of ages of the juncos in this study were selected to be balanced across populations. At the time of capture, a blood sample was collected by pricking the brachial vein with a sterile needle. For blood DNA samples collected in the field in December, about 150 μl of blood was collected into a microcapillary tube and was stored in a buffer preservative (Longmire’s solution) at 4°C until the DNA was extracted.

### Housing and sampling of birds during the captivity experiment

After capture, birds were housed briefly (1–10 days) in identical outdoor aviaries at the Mountain Lake Biological Station. On December 14, birds were transported to Bloomington, Indiana, where they were brought into an indoor aviary environment until they were resampled in early and late March. The purpose of resampling the juncos in captivity in the spring was to test the hypothesis that haemosporidian parasites go dormant in the host organs during the winter and re-emerge and start replicating in the host bloodstream during the spring. Because haemosporidian parasites are obligately vector-transmitted, and because there were no vectors in our indoor aviary environment, we assume that there was no transmission of haemosporidian parasites among juncos while they were in captivity.

In the indoor aviary environment, birds were fed *ad libitum*, and maintained on a photoperiod that was advanced every three days to match the natural photoperiod at their capture site (Mountain Lake Biological Station, VA, 37.37°N, 80.52°W). They were housed in all-male, mixed-population flocks with equal numbers of migrants and sedentary birds until February 27, when they were individually housed in cages in seven replicate rooms. Each room housed 3 migrants and 3 sedentary birds. The temperature was maintained at 16° ± 2° C [23]. Blood DNA samples for measuring haemosporidian parasites were collected again from each bird in early March (March 4–5), and again in late March (March 25–27). After collection of blood from the birds in captivity, plasma was separated from the red blood cells by centrifugation, the plasma (supernatant) was removed from the tube with a sterile Hamilton syringe, and the red blood cells were stored at -20° C until the DNA was extracted.

### Metyrapone implant treatments

For the purposes of another experiment (unpublished), unrelated to the research goals of the present study, we implanted half the birds with metyrapone, a drug that blocks the production of glucocorticoids. Our first two parasite sampling time points (December and early March) were prior to metyrapone treatment, while our final parasite sampling time point (late March) was post-metyrapone treatment. We found no effect of metyrapone on haemosporidian prevalence, or on any physiological parameter we measured, so we pooled metyrapone implant treatment birds with control birds for all analyses presented in this study (see Supporting Information for details on metyrapone treatment).

### Measuring haemosporidian parasite infection

#### DNA extractions

Genomic DNA (gDNA) was extracted from blood, using IBI Scientific MINI Genomic DNA kits (IB46701). For the December samples, about 100 μL of avian blood/Longmire’s solution from each sample was used for the DNA extractions. For the early March and late March samples, about 32.5 μl of red blood cells (after plasma had been removed) was used for the DNA extractions. gDNA was measured with a NanoDrop spectrophotometer (Take 3) and samples with insufficient quality or concentration of DNA were re-extracted prior to measuring haemosporidian infections.

#### Quantitative PCR to determine parasitemia and prevalence

We used a quantitative PCR (qPCR) to estimate the prevalence of haemosporidian parasite infections in the host populations, and the relative abundance (parasitemia) of haemosporidian parasites in the blood samples of infected birds [24]. Parasitemia levels may be predictive of the degree of pathology haemosporidian parasites are likely to cause in birds, as wild birds with higher parasitemias of some haemosporidian parasite lineages have been shown to experience greater reproductive and survival costs relative to birds with lower parasitemias [25]. We used the primers L9 5’-AAA-CAATTCCTAACAAAACAGC-3’ and NewR 5’ACATCCAATCCATAATAAAGCA-3’ [24], which target a 188-bp region of the cytochrome b (cyt b) gene. qPCR estimates of parasitemia using these primers were previously shown to be strongly positively correlated with *Plasmodium* infection status determined by a restriction enzyme-based assay [24, 26]. Our primers L9 and NewR are reported to be *Plasmodium-*specific, and in a previous study showed no association with *Leucocytozoon* diagnosis based on microscopy [24]. As far as we are aware, the sensitivity of L9 and NewR to *Haemoproteus* has not been tested.

To create the template for the standard curve, the full length haemosporidian cyt b gene from a positive sample was amplified using the primers DW2 5’-TAATGCCTAGACGTATTCCTGATTATCCAG-3’ and DW4 5’-TGTTTGCTTGGGAGCTGTAATCATAATGTG-3’ [27]. The amplicon (1356 bp) was purified, quantified, and the copy number was estimated. Serial dilutions over six orders of magnitude of this DNA were then used on each qPCR plate to create a standard curve.

DNA for qPCR was quantified using Qubit® 2.0 Fluorometer and diluted to a working concentration of 2ng/μl (10 ng of DNA was used per reaction). The qPCR absolute quantification experiment was done on Applied Biosystems® 7500 Real-Time PCR system (Life Technologies, CA, USA) using Power SYBR® Green PCR Master Mix (Life Technologies, CA, USA).

Each reaction was run in triplicate and parasite number (i.e. the number of haemosporidian cyt b gene copies per 10 ng of total DNA (host + parasite)) was estimated by calculating the mean value for the triplicate. Our qPCR estimates were not used to estimate the absolute number of parasites per bird. However, we did use our qPCR estimates to compare the relative parasitemias of haemosporidian parasites among samples. All dissociation curves were examined for the presence of nonspecific amplification or primer dimer formation. None was detected.

For the purposes of determining infection prevalence, we scored each bird with a qPCR haemosporidian copy estimate of zero as uninfected, and we scored any bird with a positive (non-zero) haemosporidian copy estimate as infected. Infection prevalence was also estimated using nested PCR (see below). Here we report both the infection prevalence results based on qPCR and the infection prevalence results based on nested PCR. Our prevalence estimates based on both methods were qualitatively similar. We only included birds for which we had parasitemia data at all three sampling time points in our analyses (i.e. we did not include birds with missing data). There were 18 migrant juncos and 19 sedentary juncos included in our statistical analyses of prevalence and parasitemia.

#### Nested PCR and sequencing to determine parasite infection prevalence, and parasite identities and evolutionary relationships

The concentration of DNA in the final extracted samples used for nested PCR ranged from 20.6–255.3 ng/μl. Haemosporidian infection was determined by amplifying the haemosporidian parasite cyt b gene using published nested polymerase chain reaction (PCR) protocols [28]. Each gDNA sample was screened for the presence of *Plasmodium* and *Haemoproteus* parasites using the external PCR primers HAEMNF and HAEMNR2 followed by the nested or internal primers HAEMF and HAEMR2 (Waldenström et al. 2004). To verify infection status of each sample, PCR was independently run twice per sample, followed by gel electrophoresis of the PCR products. A negative control (water and PCR reagents with no DNA template) was also run to confirm that there was no contamination of haemosporidian DNA in the PCR reagents. Gels were scored treatment blind by RDM and SPS to determine haemosporidian infection status and infection prevalence within each population. A sample was considered positive (infected) if there was a visible band (at 478bp) on at least one of the gel runs. Nested PCR products from positive samples were sequenced using Sanger sequencing at the Western South Dakota DNA Core Facility at Black Hills State University (BigDye v3.1 Cycle Sequencing; ABI3130 series Genetic Analyzer). Using Sequencher v5.4.6 software, the forward and reverse sequences were manually trimmed at 5’ and 3’ ends for poor quality base calls and assembled automatically to form a contiguous sequence for each sample’s PCR product. Any remaining ambiguous base calls were resolved manually. Ten sequences did not have good quality complimentary sequences on the ends and could not form contigs. These were trimmed at the ends in Sequencher to include in the project with the aligned sequences.

#### Reconstructing parasite phylogenies from parasite sequence data

**Fig 1:**
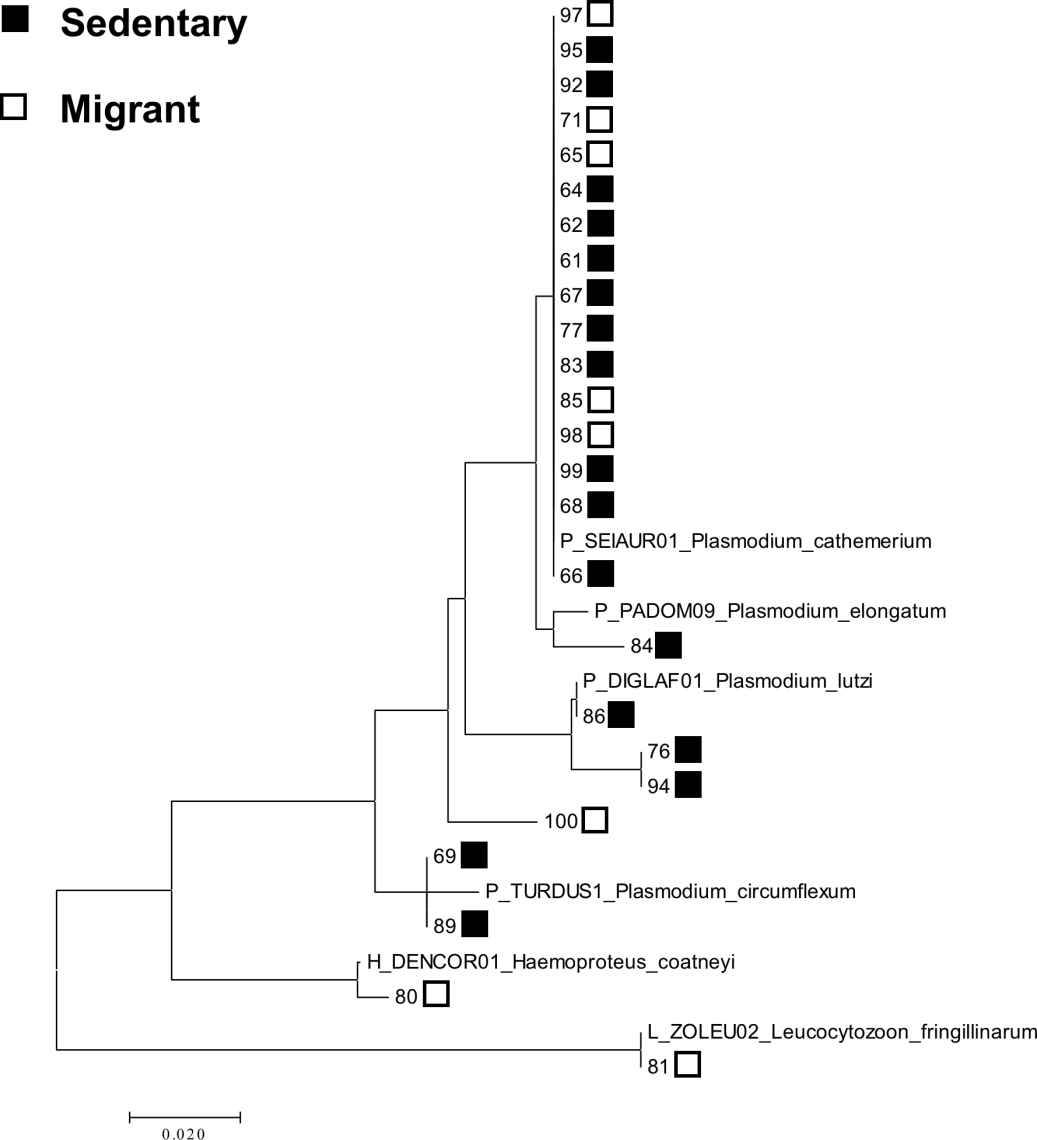
A maximum likelihood phylogeny of the haemosporidian parasites. **The phylogeny is** based on sequences from samples collected in December. Branch tips with filled boxes represent parasites sequenced from sedentary junco blood samples. Branch tips with unfilled boxes represent parasites sequenced from migrant junco blood samples. Numbers represent individual blood sample IDs. Horizontal branch length represents phylogenetic distance (substitutions/site). For reference, the parasite sequences from the described morphospecies on the MalAvi data base that most closely matched each parasite lineage in our phylogeny are included in the phylogenetic tree.

Haemosporidian parasite sequences were aligned in Molecular Evolutionary Genetics Analysis Version 7.0 (MEGA7) [29] using the ClustalW method. Following alignment, sequences were trimmed manually. The phylogeny of the parasites was inferred in MEGA using the Maximum Likelihood method based on the Tamura-Nei model [30]. Initially, a phylogenetic tree was reconstructed using the parasite sequences from all the infected juncos in the present study as well as parasite sequences from all of the described haemosporidian morphospecies on the MalAvi database [31]. Subsequently, we removed the sequences of all the morphospecies in the MalAvi database, except for the morphospecies which most closely matched the parasite sequences from the juncos in our study, which we retained for phylogenetic reference. The tree with the highest log likelihood (-907.1152) is shown (Fig 1). Initial tree(s) for the heuristic search were obtained automatically by applying Neighbor-Join and BioNJ algorithms to a matrix of pairwise distances estimated using the Maximum Composite Likelihood (MCL) approach, and then selecting the topology with superior log likelihood value. The tree is drawn to scale, with branch lengths measured in the number of substitutions per site. The analysis involved 31 nucleotide sequences. Codon positions included were 1st+2nd+3rd. All positions containing missing data were eliminated. There was a total of 320 positions in the final dataset.

### Statistical methods

95% binomial confidence intervals around infection prevalence estimates, for visualization on our figures, were computed using the online statistical calculator JavaStat http://statpages.info/confint.html. Phylogenetic analyses were performed in MEGA7. All other statistical tests were run in SPSS version 24.

#### Comparing haemosporidian prevalence between populations (objective 1) and among sampling time points (objective 2)

We used a generalized estimating equation (GEE) with United States Fish and Wildlife Service (USFW) bird band number (i.e. individual identity) as a subject variable, time point (December, early March, or late March) as a within-subject’s variable, population (sedentary or migrant) and age class (first year or after first year) as between-subjects variables. In one analysis, we used qPCR infection status (positive or negative) as our dependent variable. In a separate analysis, we used nested PCR infection status (positive or negative) as our dependent variable. We used a robust estimator for the covariance matrix with an exchangeable working correlation matrix structure, and we used a binary logistic regression for the type of model. We found no significant effect of age on haemosporidian infection status (in the qPCR analysis *P* = 0.175, in the nested PCR analysis *P =* 0.118), so we removed age from the model and report the results from analyses in which age was not included in the model. In the qPCR analysis goodness of fit QIC = 138.1. In the nested PCR analysis goodness of fit QIC = 134.2.

#### Post hoc tests

Because we found significant effects of population and of sampling time point on haemosporidian infection prevalence in the overall GEE models, we used post-hoc tests in the GEE models to assess whether there was a significant difference between infection prevalence in the sedentary versus migrant populations within each of the three sampling time points, and to test for differences in haemosporidian infection prevalence across all time point pairwise comparisons.

#### Comparing haemosporidian parasitemias between populations (objective 1)

We used a Mann-Whitney U test to determine whether average parasitemias differed between the sedentary and the migrant junco populations. For this analysis, we averaged the parasitemia score for each bird across the three sampling time points. Because we wanted to assess whether population was associated with parasitemias *within infected birds*, we excluded all the birds from both populations that were uninfected throughout the entire study from this analysis (i.e. we excluded all the birds with an average parasitemia of zero). By excluding uninfected birds, we reduced the sample size for this analysis from 19 sedentary juncos and 18 migrant juncos (all the juncos in the study) to 17 sedentary juncos and 9 migrant juncos (only the infected juncos in the study).

### Research ethics statement

All procedures involving live animals were approved by the Indiana University Institutional Animal Care and Use Committee and conducted under scientific collecting permits issued by the Virginia Department of Game and Inland Fisheries (permit 47553) and the US Fish and Wildlife Service (permit MB093279).

## Results

### Comparisons of parasite prevalence between populations (objective 1) and sampling time points (objective 2)

**Fig 2:**
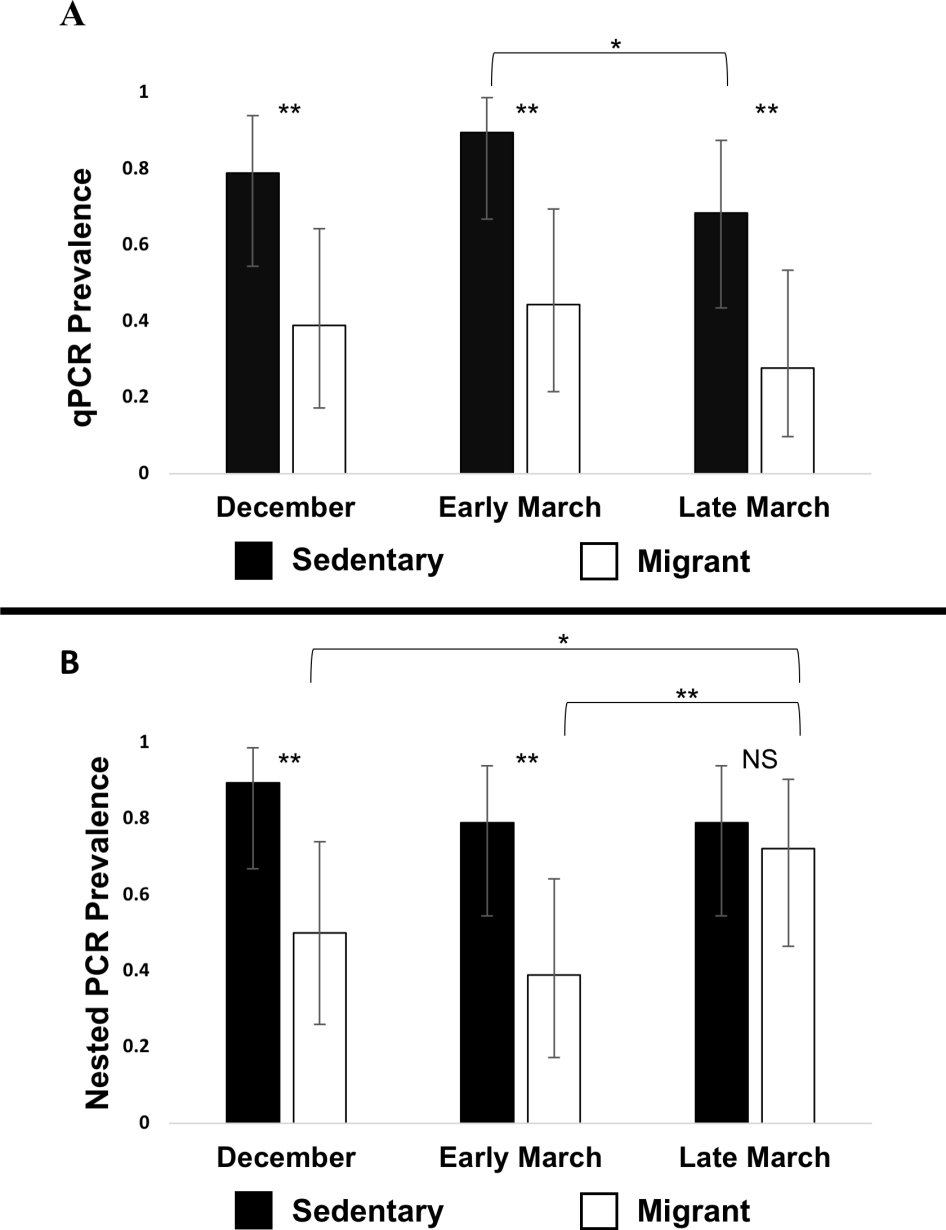
Haemosporidian infection prevalence in the blood. Prevalence estimates are based on qPCR (A) or nested PCR (B), in the sedentary population (filled bars, n = 19) and the migrant population (unfilled bars, n = 18). Error bars represent 95% binomial confidence intervals. Asterisks between filled and unfilled bars indicate significant differences between populations at each time point (* = p < 0.05, ** = p < 0.01). Brackets (top) indicate within-population cross time point comparisons where infection prevalences differed significantly (no brackets shown where infection prevalences did not differ across time points).

In the qPCR analysis, in the model including all three time points, haemosporidian infection prevalence was significantly higher in the sedentary population than in the migrant population (Generalized Estimating Equation, Wald Chi-Square_1_ = 8.666, *P* = 0.003, Fig 2A). We also found a significant effect of time point on infection prevalence (Wald Chi-Square_2_ = 7.562, *P* = 0.023, Fig 2A). We found no significant interactions between sampling time point and population (Wald Chi-Square_2_ = 0.953, *P* = 0.621). Post hoc tests analyzing infection data from each time point individually revealed that the sedentary population exhibited a significantly higher infection prevalence than the migrant population at each of the three sampling time points: December (*P* = 0.007), early March (*P* = 0.001), and late March (*P* = 0.007) (Fig 2A). Furthermore, post hoc tests also revealed that infection prevalence did not significantly change from December to early March in the sedentary population (*P* = 0.135) or the migrant population (*P* = 0.560). There was a significant decrease in infection prevalence from early March to late March in the sedentary population (*P* = 0.024) and a marginally significant decrease in infection prevalence from early March to late March in the migrant population (*P* = 0.058) (Fig 2A). Overall, the infection prevalence did not change significantly from the beginning of the study (December) to the end of the study (late March) in the sedentary (*P* = 0.304) or the migrant (*P* = 0.303) population.

**Fig 3:**
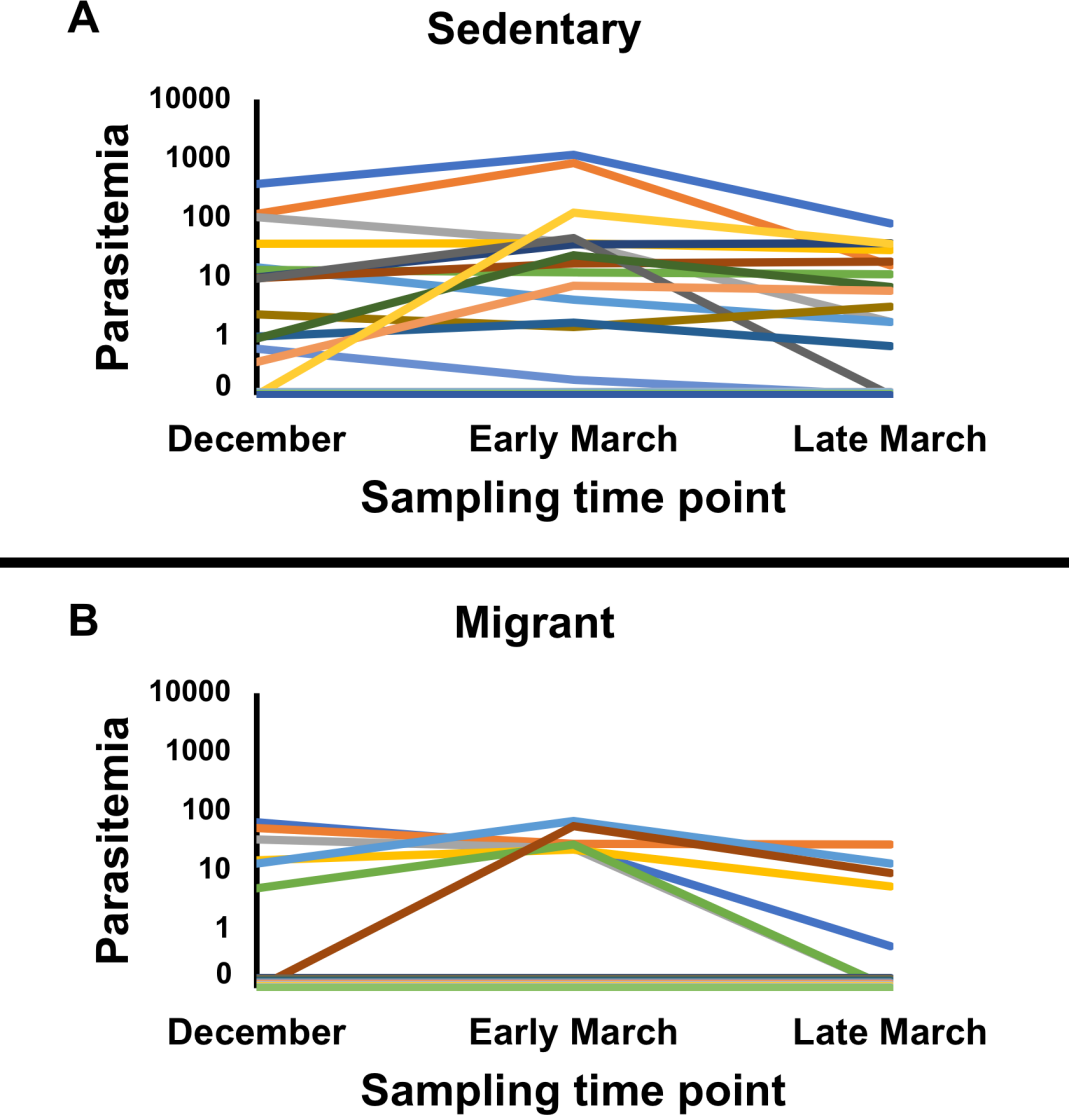
Haemosporidian parasitemias of individual juncos. Haemosporidian parasitemias (the number of haemosporidian cyt b gene copies per 10 ng of total DNA (host + parasite), as determined by qPCR) in the bloodstream of individual sedentary (panel A, n = 19) and migrant (panel B, n = 18) juncos across time points. Each colored line represents an individual junco. Overlapping lines representing uninfected birds are stacked on top of each other at the bottom for visualization.

In the nested PCR analysis, in the model including all three time points, haemosporidian infection prevalence was significantly higher in the sedentary population than in the migrant population (Generalized Estimating Equation, Wald Chi-Square_1_ = 4.364, *P* = 0.037, Fig 2B). We found no significant effect of time point on infection prevalence (Wald Chi-Square_2_ = 4.560, *P* = 0.102, Fig 2B). We found a marginally significant interaction between sampling time point and population (Wald Chi-Square_2_ = 6.210, *P* = 0.045). Post hoc tests analyzing infection data from each time point individually revealed that the sedentary population exhibited a significantly higher infection prevalence than the migrant population in December (*P* = 0.004) and early March (*P* = 0.007), but there was no difference in infection prevalence between the two populations in late March (*P* = 0.633) (Fig 2B). Furthermore, post hoc tests also revealed that infection prevalence did not significantly change from December to early March in the sedentary population (*P* = 0.135) or the migrant population (*P* = 0.134). There was no significant change in infection prevalence from early March to late March in the sedentary population (*P* = 1.0) but there was a significant increase in infection prevalence from early March to late March in the migrant population (*P* = 0.003) (Fig 2B). Overall, the infection prevalence did not change significantly from the beginning of the study (December) to the end of the study (late March) in the sedentary population (*P* = 0.135) but did significantly increase in the migrant population (*P* = 0.023). The parasitemias of individual juncos over time can be visualized in Fig 3.

### Relationship between host population and parasitemias of infected birds

**Fig 4:**
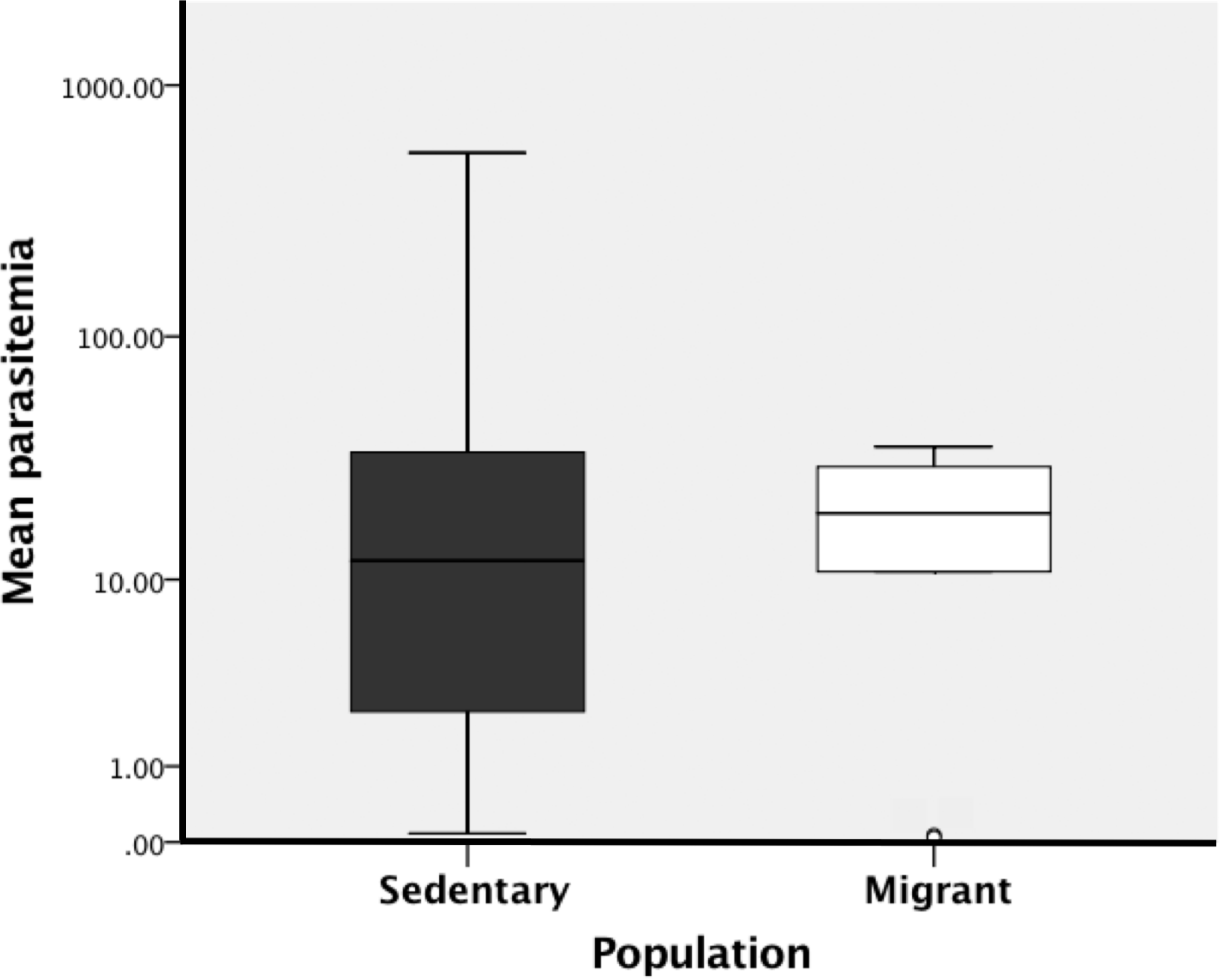
Boxplot of mean parasitemias. The mean parasitemias (the number of haemosporidian cyt b gene copies per 10 ng of total DNA (host + parasite), as determined by qPCR, averaged across the three sampling time points: December, early March, and late March) of sedentary (filled, n = 17) juncos and migrant (unfilled, n = 9) juncos. Only infected juncos were included in this analysis. The horizontal lines inside the boxes represent the sample medians. The length of the box represents the interquartile range. Whiskers span all the data except for statistical outliers. Statistical outliers are points that are at least 1.5 box lengths away from the edge of the box.

When considering only birds that were infected during at least one sampling time point during the study (average parasitemia > 0), we found no difference between the medians of the average parasitemia (averaged across the three sampling time points in the study) in the sedentary versus the migrant population (*U* = 77, *z* = 0.027, *p* = 1.0, Fig 4).

### Community composition of the haemosporidian parasites

We considered parasite cyt b sequences to belong to different lineages when they differed by at least one base pair. We detected five distinct haemosporidian parasite lineages (sequenced from 17 infected birds) in the sedentary population. We detected four distinct haemosporidian lineages (sequenced from eight infected birds) in the migrant population. One lineage (SEIAUR01; *Plasmodium cathemerium*) was detected in both populations and was also the most prevalent lineage detected within each population. All of the lineages detected within the sedentary population belonged to the genus *Plasmodium*. The genera *Plasmodium*, *Haemoproteus*, and *Leucocytozoon* were all detected in the migrant population.

## Discussion

### Objective 1: Association between host migration and infection prevalence and parasitemia

We found that a sedentary population of dark-eyed juncos maintains a higher prevalence of haemosporidian parasite infections, relative to a closely related conspecific migrant population, both when sampled during seasonal sympatry on shared wintering grounds in the Appalachian Mountains of Virginia (December), and again at two early spring time points (early March and late March) during study in a captive common environment. Among infected juncos, we found no difference between the parasitemias circulating in the bloodstream of sedentary versus migrant juncos. These results are consistent with the migratory culling hypothesis and with the migratory escape hypothesis and suggest that long-distance migration may reduce the prevalence of haemosporidian parasites in birds. The higher prevalence of parasites that we observed in sedentary hosts is also consistent with previous empirical research showing that migration is associated with reduced parasitism in monarch butterflies [8, 9] and galaxiid fishes [7].

On the other hand, previous research in another avian blood-parasite system demonstrated a very different relationship between host migration and parasitism. Kelly, MacGillivray [16] found that longer-distance adult song sparrow migrants were *more* likely, on average, to be infected with blood-borne parasites (primarily *Plasmodium*, *Haemoproteus*, and *Leucocytozoon*) than sparrows that migrated shorter distances. It is unclear why we found that long-distance migration is associated with reduced parasitism in juncos, while Kelly, MacGillivray [16] found the opposite pattern in a closely related host. Future research should investigate why the association between long-distance migration and parasitism differs across study systems and ecological contexts.

Migratory culling and migratory escape are non-mutually exclusive mechanisms that each could explain the reduced prevalence of haemosporidian parasites that we observed in migrant juncos. Within our data set, it is not possible to eliminate either of these potential mechanisms. Future research should assess the possible contributions of each mechanism to the population difference that we observed by testing additional predictions made by the migratory culling and migratory escape hypotheses. For example, the migratory culling hypothesis predicts that parasites reduce flight performance and/or increase the costs of long-distance flight. This prediction has received support in butterflies infected with a protozoan parasite [5], but did not receive support in a study of great reed warblers infected with *Plasmodium* parasites [32]. A comparison of the seasonal density profiles of (infected) vectors at the breeding site for sedentary juncos (Mountain Lake Biological Station in Virginia) versus at breeding sites for migrant juncos (in Alaska, Canada, and/or Northern New England) could provide a more direct test for the migratory escape hypothesis, as the migratory escape hypothesis predicts that migrant birds should experience reduced haemosporidian parasite exposure at their breeding grounds relative to sedentary birds.

We reject migratory exposure and migratory susceptibility as primary drivers of variation in infection prevalence between our study populations. The migratory exposure hypothesis and the migratory susceptibility hypothesis predict that sedentary host populations should exhibit *lower* parasite infection prevalence relative to closely related migrant populations. Therefore, our observation that a sedentary host population exhibited a *higher* prevalence of haemosporidian parasite infections directly contradicts a key prediction of these hypotheses.

Our analysis of infection prevalence based on qPCR produced qualitatively similar results in December and early March to our analysis based on nested PCR (Fig 2). In both analyses sedentary birds exhibited higher infection prevalence than migratory birds in December and early March. However, our analyses of infection prevalence produced qualitatively different results in late March. Our qPCR results suggest that the sedentary population maintained a significantly higher haemosporidian infection prevalence than the migratory population in late March. However, our nested PCR results suggest that the infection prevalence in the migratory population increased between early March and late March, resulting in no significant population difference in infection prevalence in late March. We do not know why our metrics of infection prevalence produced different results in late March. Perhaps differences in the sensitivity and/or specificity of the qPCR and nested PCR primers could explain this discrepancy in our results. The nested PCR primers we used are sensitive for detecting both *Haemoproteus* and *Plasmodium* infections in avian blood [33]. The qPCR primers that we used are sensitive for detecting *Plasmodium* but not *Leucocytozoon* infections, and the sensitivity of our qPCR primers to *Haemoproteus* is unknown [24]. Overall, taking data from all three time points together, both methods (qPCR and nested PCR) provided support for the prediction that sedentary juncos exhibit higher haemosporidian infection prevalence relative to migratory juncos at our study site.

### Community composition of the haemosporidian parasites

While five distinct haemosporidian lineages were detected in the sedentary population and four distinct haemosporidian lineages were detected in the migrant population, one haemosporidian lineage (SEIAUR01; *Plasmodium cathemerium*) was dominant in both our sedentary and migrant populations. This dominance of the same parasite lineage in both populations suggests that haemosporidian transmission may occur between the sedentary and migrant populations. Alternatively, it is possible that *Plasmodium cathemerium* was dominant in both populations because it is a common and widespread lineage that was encountered and acquired independently by the sedentary and migrant populations at their respective breeding grounds. Sharing of haemosporidian parasite lineages between seasonally sympatric sedentary and migratory host populations has been previously observed in other avian systems. Clark, Clegg [34] found that some haemosporidian parasite lineages were shared between wintering migrant and resident wading birds in Australia at their shared wintering grounds, suggesting active transmission between migrants and residents. Ricklefs, Medeiros [35] found that parasite lineage sharing was more likely to occur between sedentary and migrant birds sharing habitat when the sedentary and migrant birds were more taxonomically similar.

### Objective 2: Examining seasonality in the parasite lifecycle

Contrary to our expectations, we found no evidence for winter dormancy of the haemosporidian parasites. A substantial prevalence of haemosporidian parasite infections was detectable (via qPCR and nested PCR methodology) in the blood stream in midwinter (December) in both host populations, suggesting that haemosporidian parasites do not markedly sequester and go dormant in the host organs in our study system. We found mixed evidence for a spring re-emergence of haemosporidian parasites from the host organs into the host bloodstream in the early or late March sampling points, as neither the prevalence nor parasitemia of haemosporidian infections increased over the course of the study in either population in our qPCR analysis, however, in our nested PCR analysis infection prevalence increased over the course of the study in the migrant but not in the sedentary population.

We propose three possible explanations for why we did not observe a detectable increase in the prevalence or parasitemia of haemosporidian parasites in the bloodstream from December to late March in our qPCR analysis, and for why we only detected an increased infection prevalence in the migrant but not in the sedentary population in our nested PCR analysis. First, it is possible that the pattern of dormancy in the organs during the winter, followed by replication in the bloodstream during the spring and summer, may not occur in our study populations. The evidence for winter dormancy of haemosporidian parasites in temperate avian populations is mixed. Some previous studies have found a pattern of high levels of haemosporidians circulating in the avian blood stream during the spring and summer, with low or undetectable parasite levels during the winter [36–38], suggesting a pattern of winter dormancy. On the other hand, another study found a high haemosporidian prevalence detectable in the bloodstream of rusty blackbirds (*Euphagus carolinus*) during the winter in Mississippi and Arkansas, suggesting that winter dormancy of haemosporidian parasites may not occur in those populations [39]. Consistent with the Barnard, Mettke-Hofmann [39] study, our results suggest that winter dormancy in the host organs may not occur in some temperate avian haemosporidian populations.

Alternatively, while the high prevalence of infections that we measured in December clearly demonstrates that haemosporidians are still circulating in the blood stream during the winter, and are not completely sequestered in the host organs, it is still possible that haemosporidians in our study system exhibit partial dormancy and sequestration in the organs. Therefore, we propose that a second possible explanation for why we did not observe a stronger pattern of seasonal increase in parasite prevalence or parasitemia over the course of our study is that not all of the seasonal environmental cues required to induce spring re-emergence of haemosporidians into the host bloodstream were present in the indoor aviary environment where the juncos were housed in our study. The juncos in our study experienced a lengthening photoperiod, set to match the natural photoperiod at their capture site in Virginia. This lengthening photoperiod of the common aviary environment was previously shown to be a sufficient cue of seasonal change to induce reproductive (physiological and behavioral) development in the sedentary population [23]. However, a longer photoperiod than the birds experienced in late March (the end of our study) may be required to induce the re-emergence of haemosporidians into the blood stream. Alternatively, lengthening photoperiod may not be a sufficient environmental cue, on its own, to induce parasites to re-emerge from dormancy in the host organs and to start replicating in the bloodstream. In addition to photoperiodic cues, the activity and replication of avian haemosporidian parasites may respond plastically to supplemental cues such as changes in temperature, food availability, or exposure to vectors [40].

Finally, the effect of maintaining birds in an indoor aviary environment on haemosporidian parasite infections is unknown. It is possible that the stress of being maintained in an indoor aviary environment could lead to year-round chronic infection. To our knowledge, no previous studies have measured seasonal activity of avian haemosporidian parasites in a controlled indoor aviary environment. Future research should manipulate photoperiod in combination with manipulations in supplemental cues in a controlled indoor aviary environment to assess whether lengthening photoperiod, supplemental cues, or a combination of lengthening photoperiod and supplemental cues can induce the re-emergence and replication of dormant haemosporidian parasites.

## Conclusions

In the present study, we provide evidence that sedentary juncos maintain a higher prevalence (but not parasitemia) of haemosporidian parasite infections throughout the winter and early spring relative to migrant juncos in a seasonally sympatric population. Our results suggest that long-distance migration may reduce the prevalence of avian haemosporidian parasites, although since we only compared 2 populations (one migratory and one sedentary) we cannot rule out the possibility that ecological differences between the populations other than migration (e.g. stochastic variation in abundance of parasites and/or vectors at the allopatric breeding grounds) could explain the observed population differences in parasite prevalence. Migratory culling and migratory escape are non-mutually exclusive alternative mechanisms that could potentially explain the observed lower infection prevalence in migrants. We are unable to eliminate either of these as potential mechanisms. We reject migratory exposure and migratory susceptibility as primary mechanisms explaining differences in haemosporidian infection prevalence between our study populations. Also, we found no evidence that haemosporidian parasites in our study system exhibit dormancy and sequestration in the host organs during the winter. Future research should compare haemosporidian infections across more sedentary and migrant populations within a vertebrate host species, to assess whether migrant populations consistently exhibit a lower infection prevalence, as we observed. Additionally, future research should compare the diversity of parasites infecting sedentary versus migrant conspecific host populations to test the Migratory Exposure hypothesis’ prediction that migrant host populations accumulate more diverse parasite communities. Additionally, future research should measure the density of (infected) vectors across the breeding and non-breeding ranges of sedentary and migrant populations to assess how long-distance migration affects exposure to haemosporidian parasites. Finally, future research should assess whether female juncos, and juvenile juncos, exhibit the same relationship between long-distance migration and parasitism that we observed in adult males. Overall, our research suggests that long-distance migration may reduce parasitism in migrating hosts.

## Supporting information

S1 Text (DOCX)

S1 Fig (TIFF)
