# Supplementary material for: Sedentary songbirds maintain higher prevalence of haemosporidian parasite infections than migratory conspecifics during seasonal sympatry
Source: PLoS One. 2018 Aug 22;13(8):e0201563. doi: 10.1371/journal.pone.0201563 (PMC6104930; doi:10.1371/journal.pone.0201563)
Supplement: S1 Text — (DOCX) [file pone.0201563.s001.docx]

**Supporting Information**

**Metyrapone implant treatments**

For the purposes of another experiment (unpublished), unrelated to the research goals of the present study, we implanted half the juncos with metyrapone, a drug that blocks the production of glucocorticoids. On March 4, 2014, surgical scissors were used to make a small (~2.5 mm) opening in the skin along the left flank of each bird. Sterile 10 mm silastic tubing (1.5mm i.d. 2.0 o.d.; Dow Corning) either empty (control) or packed with metyrapone (Sigma Aldrich, M-2696) and sealed were inserted in the opening and gently pushed perpendicular to the hole to ensure that they did not come out before healing. Birds were inspected weekly to ensure that implants had not fallen out. On March 16, 2014, metyrapone implants were removed and replaced. Control birds were handled for the same amount of time as birds that received metyrapone implants, as a sham procedure.

We used a generalized estimating equation to test the effects of population, sampling time point, metyrapone implant treatment, and population*time point interaction and implant treatment*time point interaction on haemosporidian parasite infection prevalence. Goodness of fit QIC = 141.346. We found a significant effect of population (Wald Chi-Square_1_ = 9.741, p = 0.002). We found a significant effect of time point (Wald Chi-Square_2_ = 7.850, p = 0.020). We found no significant time point by population interaction (Wald Chi-Square_2_ = 1.566, p = 0.457). We found no significant effect of implant (Wald Chi-Square_1_ = 1.081, p = 0.298). We found no significant time point by implant interaction (Wald Chi-Square_2_ = 0.785, p = 0.675). Because we found no effects of metyrapone implants on haemosporidian infections, and because the relationships between population, time point, and parasite prevalence were not qualitatively different when we included or excluded metyrapone treatment from our analysis, we pooled metyrapone implant treatment birds with control birds, and did not include metyrapone treatment in the analyses presented in this study. The prevalence of haemosporidian parasite infections within each metyrapone implant treatment can be seen in S1 Fig.

**Fig. S1.** Haemosporidian infection prevalence in the sedentary population (red) and the migrant population (blue). Control implant treatment birds are represented by solid bars and metyrapone implant treatment birds are represented by hashed bars.
