# Supplementary figures and images for: Sedentary songbirds maintain higher prevalence of haemosporidian parasite infections than migratory conspecifics during seasonal sympatry
Source: PLoS One. 2018 Aug 22;13(8):e0201563. doi: 10.1371/journal.pone.0201563 (PMC6104930; doi:10.1371/journal.pone.0201563)

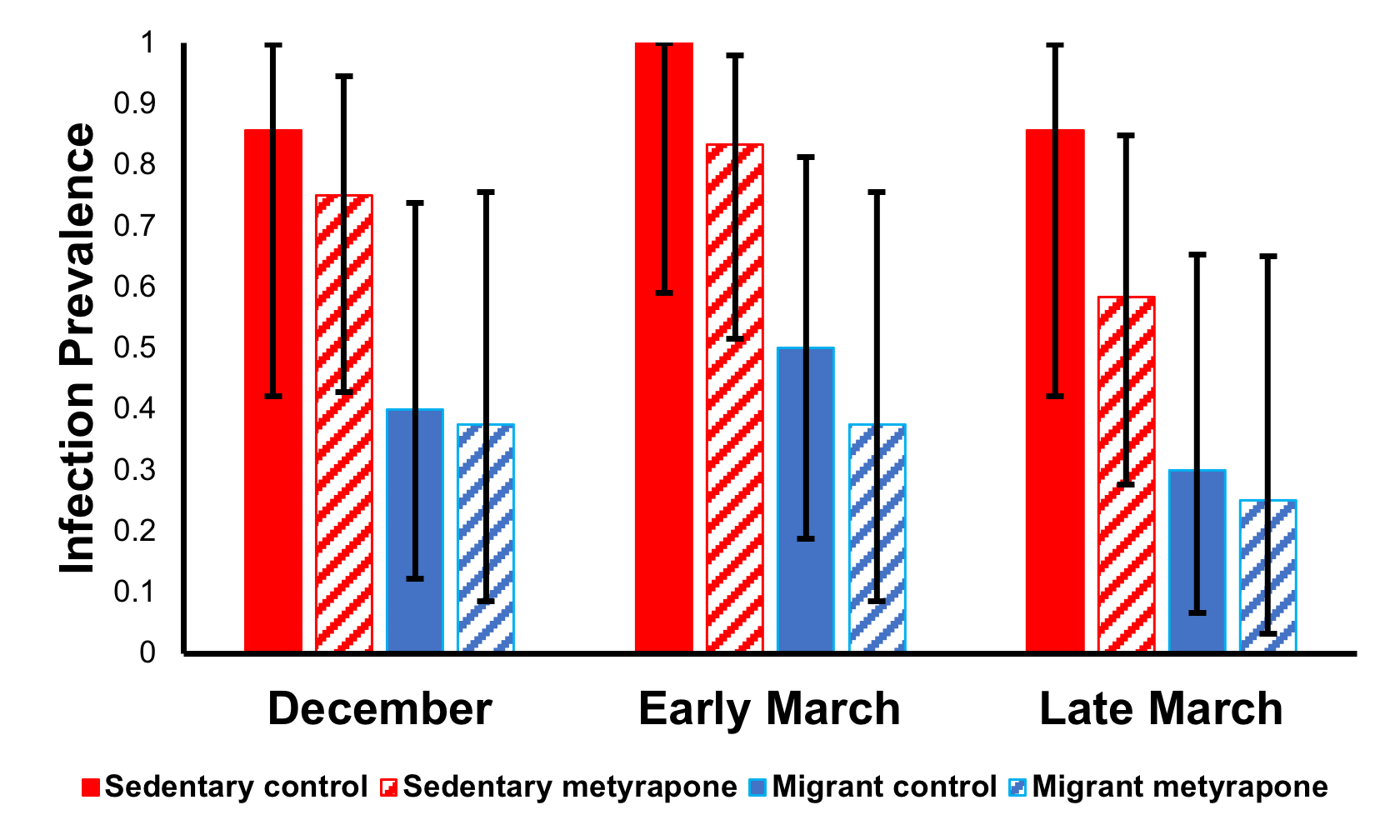

Supplement: S1 Fig — (TIFF) [file pone.0201563.s002.tiff]
